# Passive Smoking in the Etiology of Non-Syndromic Orofacial Clefts: A Systematic Review and Meta-Analysis

**DOI:** 10.1371/journal.pone.0116963

**Published:** 2015-03-11

**Authors:** Heba J. Sabbagh, Mona Hassan Ahmed Hassan, Nicola P. T. Innes, Heba M. Elkodary, Julian Little, Peter A. Mossey

**Affiliations:** 1 Pediatric Dentistry Department, Faculty of Dentistry, King Abdulaziz University, Jeddah, Saudi Arabia; 2 Department of Oral Health Sciences, University of Dundee, Dundee, Scotland, United Kingdom; 3 Department of Dental Public Health, Faculty of Dentistry Kind Abdulaziz University, Jeddah, Saudi Arabia; 4 Department of Biostatistics, High Institute of Public Health, Alexandria University. Alexandria, Egypt; 5 Paediatric Dentistry, Department of Oral Health Sciences, University of Dundee, Dundee, Scotland, United Kingdom; 6 Faculty of Dental Medicine (Girls’ Branch), Al Azhar University, Cairo, Egypt; 7 Human Genome Epidemiology, School of Epidemiology, Public Health and Preventive Medicine, University of Ottawa, Ottawa, Ontario, Canada; 8 Division of Oral Health Sciences and WHO Collaborating Centre for Oral Health & Craniofacial Anomalies, University of Dundee, Dundee, Scotland, United Kingdom; Medical University of South Carolina, UNITED STATES

## Abstract

**Background:**

Studies have found a consistent positive association between maternal smoking and non-syndromic orofacial clefts (NSOFC). However, no comprehensive assessment of the association between NSOFC and passive smoking has been undertaken. This systematic review and meta-analysis explores the relationship between maternal passive smoking and NSOFC, and compares the associations between passive and active smoking.

**Methods and Findings:**

Search strategy, inclusion / exclusion criteria, and data extraction from studies reporting maternal passive smoking and NSOFC was implemented without language restrictions. Risks of bias in the identified studies were assessed and this information was used in sensitivity analyses to explain heterogeneity. Meta-analysis and meta-regression of the extracted data were performed. Egger's test was used to test for small study effects. Fourteen eligible articles were identified. Maternal passive smoking exposure was associated with a twofold increase in risk of NSOFC (odds ratio: 2.11, 95% confidence interval: 1.54–2.89); this was apparent for both cleft lip with and without palate (OR: 2.05, 95% CI: 1.27–3.3) and cleft palate (OR: 2.11, 95% CI: 1.23–3.62). There was substantial heterogeneity between studies. In the studies that provided data enabling crude and adjusted odd ratios to be compared, adjustment for potential confounders attenuated the magnitude of association to about a 1.5-fold increase in risk.

**Conclusion:**

Overall, maternal passive smoking exposure results in a 1.5 fold increase in risk of NSOFC, similar to the magnitude of risk reported for active smoking, but there is marked heterogeneity between studies. This heterogeneity is not explained by differences in the distribution of cleft types, adjustment for covariates, broad geographic region, or study bias/quality. This thorough meta-analysis provides further evidence to minimize exposure to environmental tobacco smoke in policy making fora and in health promotion initiatives.

## Introduction

Today’s best evidence suggests that non-syndromic orofacial clefts (NSOFC) are multifactorial in origin involving both genetic and environmental risk factors [1]. Better understanding of the etiology of environmental factors can provide the basis for prevention through avoidance of exposure to risk factors.

Previous studies have been consistent in finding a positive association between active maternal smoking and NSOFC [2]. A meta-analysis has suggested a modest positive association between active smoking and NSOFC; for cleft lip with or without cleft palate (CL/P) the relative risk was 1.34 (95% CI: 1.25 to 1.44) and for cleft palate (CP) relative risk was 1.22 (95% CI: 1.10 to 1.35) [3]. Avoidance of smoking to reduce this risk is a common public health message [4]. However, the risk of maternal smoking exposure may be underestimated, as non-smoking pregnant women might still be exposed to passive smoking (environmental tobacco exposure) at home or work [5] and this is not usually taken into account, especially in developing countries. The 2014 Surgeon General’s Report highlights a wide range of acute and chronic adverse health effects in infants and increased risk of adverse health outcomes resulting from second hand smoking [6]. The Report also noted that tobacco control measures are not sufficient to end the tobacco epidemic. Furthermore, although the association between passive smoking and congenital anomalies has been studied, the relationship has not been found to be consistent [7], and no comprehensive assessment of the association between NSOFC and maternal passive smoking exposure has been undertaken. This systematic review and meta-analysis (1) in non-smoking mothers, assesses the relationship between maternal passive smoking and having an infant with NSOFC; and (2) for all mothers in the included studies, compares the associations between maternal passive smoking and maternal active smoking. We propose that through confirmation of this relationship we will inform public health messages and support planning of community awareness programs around the adverse consequences of passive smoking during pregnancy, which is likely to be especially relevant to developing countries.

## Materials and Methods

### Search strategy and data extraction

**Fig 1:**
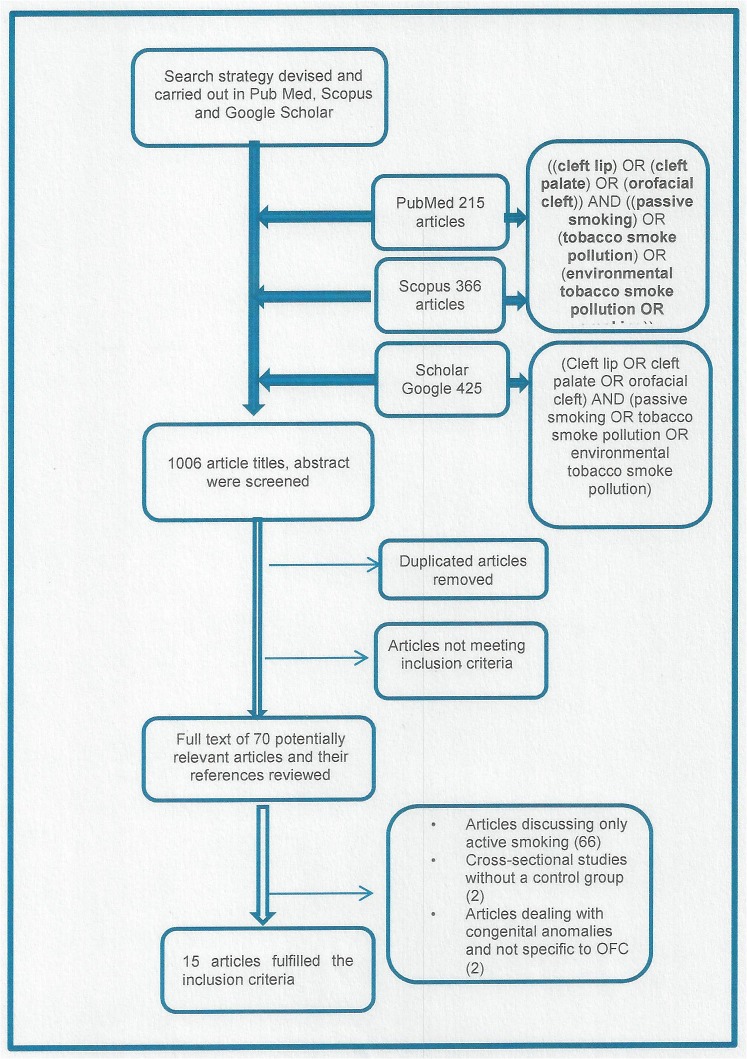
Flow diagram of study selection process.

We prepared a research protocol to investigate the relationship between orofacial clefts and passive smoking, defined as maternal exposure to environmental tobacco smoke in any location at any time during the pregnancy. The search strategy comprised key words listed separately and in combination; ((cleft lip) OR (cleft palate) OR (orofacial cleft)) AND ((passive smoking) OR (tobacco smoke pollution) OR (environmental tobacco smoke pollution) OR (smoking)). These key words were run in three search engines (PubMed, Scopus, Scholar Google) from 1980 to 2013. For Google Scholar, the search yielded 1140 articles, too numerous for review. Therefore, a modification was carried out by pooling titles from key word combinations of; ((cleft lip) OR (cleft palate) OR (orofacial cleft)) AND ((passive smoking) OR (tobacco smoke pollution) OR (environmental tobacco smoke pollution)). This revised search strategy gave a total of 1,006 articles across the three search engines: PubMed (215), Scopus (366), Google Scholar (425), (see Fig. 1). The searches were run in March 2013 and did not exclude any languages. Full details of the search strategy are available in S1 Fig.

The titles of all articles were reviewed by two authors independently (HJ and HK). Data presented more than once were excluded. Abstracts of articles selected on the basis of their titles were then reviewed. Articles were excluded where it was obvious from the title or abstract that the paper did not discuss the relationship of maternal passive smoking with NSOFC. The references of remaining articles were reviewed, then full texts were screened according to the following inclusion criteria;

- studies reporting passive smoking and its relationship to NSOFC; and
- case-control, cohort or cross-sectional studies where there was a control or comparison group.

*The exclusion criteria were*:

- studies that discussed smoking as a whole but did not provide information on the specific association between passive smoking and NSOFC;
- studies that reported associations with genes reported to modulate the effect of smoking or gene-environmental joint effects related to NSOFC but which did not report the marginal effect of passive smoking; and
- studies that included syndromic clefts in which data on non-syndromic clefts could not be extracted.

*Data extracted from these studies included*:

- passive smoking definition;
- study design and setting;
- sample size, description and base population;
- prevalence and intensity of maternal passive smoking prior to pregnancy and in the first trimester for cases and controls;
- the frequency of maternal passive smoking for cleft lip with or without cleft palate (CL/P) and cleft palate (CP). Reported odds ratios (OR) and confidence intervals (CI) without frequencies were also considered; and
- prevalence and intensity of active maternal smoking.

Data were extracted, using a data extraction form (S1 Table), independently by two authors (HJ and HK). Any disagreement was resolved by discussion with a third author (MH). When possible, authors of included studies were contacted for further information on the topic. We received a response from three authors [8–10].

### Assessing risk of bias

The quality of included articles was assessed independently by two of the authors using the Newcastle-Ottawa Scale (NOS) [11]. The scale measures three items; selection of cases and controls including their definition and representativeness; comparability of cases and controls in design and analysis; and exposure ascertainment. The scale has a minimum score of 0 and a maximum score of 9. Studies scoring 6 or more (correspond to 67% of the maximum score) were regarded as having a low risk of bias ("good" quality) [12]; 3–5 a modest risk of bias ("fair" quality); and studies <3 were considered to be at substantial risk of bias ("poor" quality) Spearman's rank correlation coefficient was used to measure the degree of agreement between the authors' judgments. Disagreements were resolved through discussion. No exclusion based on risk of bias was performed. Studies were further classified into those at substantial to modest risk of bias versus those at low risk for sensitivity analysis. Details of study quality are presented in S2 Fig.

### Consideration of possible small study effects

We used funnel plots to visually assess the possibility of small study effects for all studies together and also for those assessing the relationship between passive smoking and NSOFC phenotype (CL/P and CP) [13]. In addition, Egger’s test was used to test for small study effects.

### Statistical analysis

Meta-analysis was performed using the free software Review Manager (Cochrane Collaboration) [14]. The Mantel-Haenszel method was used for combining studies to calculate summary ORs and 95% CIs for passive smoking versus no smoking. To decide whether the results of the separate studies could be combined meaningfully, a statistical test of homogeneity was carried out. Based on the chi-square test, an inconsistency coefficient was computed (I^2^ statistic) where a value more than 50% indicated moderate, and greater than 75% indicated high, heterogeneity [15]. Odds ratios were pooled with a fixed effect model for homogeneous studies and a random effects model for heterogeneous studies. Odds ratios with their 95% confidence limits for the individual studies and summary estimate of effect were graphically displayed in a forest plot.

For comparing the result of crude OR with the reported adjusted OR, meta-analysis was carried out using OR and standard error (SE) values that were estimated from the 95% CI.

Meta-DiSc version 1.4 (http://www.hrc.es/investigacion/metadisc_en.htm) was used to perform meta-regression for assessing the possible effect of study quality and type of cleft on the relationship between passive smoking and NSOFC. Inverse variance weights and restricted maximum likelihood estimation were used.

### Sensitivity analysis

To assess stability of the results, subgroup analyses were carried out based on (a) type of NSOFC (CL/P and CP), (b) study risk of bias (NOS score >6 vs. ≤6), (c) reported adjusted OR compared to crude OR, (d) periods of measured maternal passive smoking exposure (1^st^ trimester including and not including the pregestation period, or the pregestation period alone), (e) sequential exclusion of studies with ORs for the association between NSOFC and passive smoking greater than 3, and (f) broad geographic region in which the study was carried out (China, US and Europe, Other). These areas of subgrouping were considered likely sources of heterogeneity.

## Results

**Fig 2:**
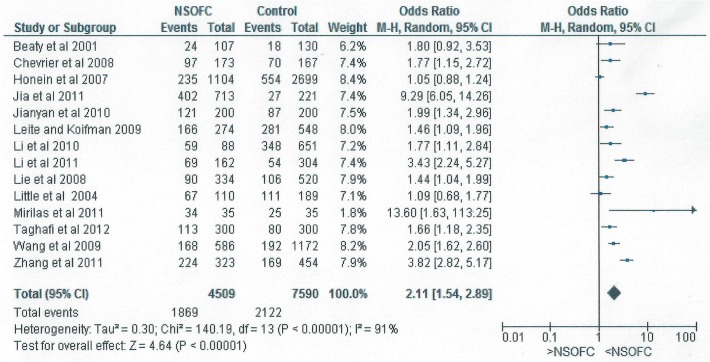
Forest plot for meta-analysis of the association between maternal passive smoking and the risk of having an infant with NSOFC.

The searches yielded 1,006 potentially eligible titles. After removing duplicate articles and reviewing the abstracts, the full text of 70 articles were obtained and compared to the inclusion criteria. Fifty-five articles were excluded (Fig. 1) because they; did not include control group (two articles), discussed congenital anomalies in general (two articles) or did not study passive smoking (51 articles). This resulted in 15 eligible articles (Table 1 and Fig. 2) [8–10, 16–27]. These were all retrospective case-control studies using self-report questionnaires for non-smoking mothers.

**Table 1:** Characteristics of studies of risk of NSOFC in the offspring of non-smoking mothers exposed to passive smoking included in meta-analysis.

Reference	Site and Country	Duration of data collection	Study design	Total Sample size (smoking + non-smoking) mothers	Reported period of maternal exposure	Non-Smoking mothers exposed to passive smoking / total non-smoking mothers (%)			Reported adjusted OR (95% CI) for passive smo0king with adjusted factors	Active smoking mothers/total sample size (%)	
						NSOFC	Type of NSOFC (CL/P and CP)	Controls		Cases	Control
**Beaty *et al* (2001)[16]**	Treatment centers, Maryland craniofacial clinics, Children's National Medical Centre, Washington DC, US	1992–1998	Case-control	171 cases	1st trimester	24/107 (22.4)	CL/P: 14/73 (19.2)	18/130 (13.8)	CL/P: 1.04 (0.067–1.62)	27/171 (15.8)	25/182 (13.7)
				182 control			CP: 10/34 (29.4)		CP: 1.17 (0.68–2.02)		
									Maternal age and education		
**Chevrier *et al* (2008)[17]**	Maxillofacial departments in; Lyon; Grenoble; Rhone-Alpes region; Paris; Clermont-Ferrand; Auvergne **France**	1998–2001	Matched case- control (age, sex, origin, place of residence)	240 cases	1st trimester	97/173 (56.1)	CL/P: 65/119 (54.6)	70/167 (41.9)	1.8 (1.2–3.4) Region and Child sex	67/240 (27.9)	69/230 (30)
				236 controls			CP: 32/54 (59.3)				
**Honein *et al* (2007)[18]**	Coordinated by the Centers for Disease Control and Prevention (CDC). Eight Centers for Birth Defects Research and Prevention contributed data: Arkansas, California, Iowa, Massachussetts, New Jersey, New York, Texas, and CDC (Atlanta, GA), **US**		Population based, multicenter case-control (site, frequency of births per month)	933 CL/P 528 CP	Three month pregestation + 1st trimester	235/1104 (21.3)	CLP/P: 147/699 CP: 88/528 (22)	554/2299 (20.5)	1.1 (0.09–1.3) Child sex, folic acid exposure, maternal age, ethnicity, gravidity	352/1461 (24.1)	684/3390 (20.2)
			Random sample of live births in controls	3390 control	Pregestation	14/1104 (1.3)	CL/P: 7/699 (1) CP: 7/405 (1.7)	21/2699	1st degree relative with birth defect were excluded		
					1st trimester	13/1104 (11.4)	CL/P: 9/6991 (0.1) CP: 4/405 (0.99)	39/2699 (1.4)			
**Jia *et al* (2011)[19]**	West China College of Stomatology, Sichuan University, Department of Cleft Lip and Palate Surgery, **China**	2008 and 2010	Hospital based, Case—control	537 CL/P 176 CP 221 controls	1st trimester	402/713 (56.2)	CL/P: 302/537 (56.2)	27/221 (12.2)	11.42 (6.87–19) Child sex, birth weight, maternal age and weight, multi-vitamins, calcium and folic acid exposure	18/713 (2.5)	2/221 (0.9)
							CP: 100/176 (56.8)				
**Jianyan *et al* (2010)[20]**	**China**		Hospital-based, matched case-control (sex, age, socio-econimic status)	200 CL/P 200 controls	Three month pregestation + 1st trimester	121/200 (60.5)		87/200 (43.5)	1.72 (1.08–2.74) Maternal and paternal schooling	a	a
**Leite and Koifman (2009)[21]**	City of Rio de Janeiro **Brazil**		Hospital-based, matched case-control (sex, age, location of parents resident)	274 cases 548 controls	One year pregestation + 1st trimester	166/274 (60.6)		281/548 (52.3)	1.48 (1.09–2.01) Maternal education, age and alcohol intake	68/274 (24.8)	94/548 (17.1)
**Li *et al* (2010)[22]**	Data from a population-based case-control study of external malformations in 4 couties (Pingding, Xiyang, Taigu, Zezhou) of Shanxi Province,38 **China**	2003–2006	Population-based Matched case-control (county, sex, maternal ethnic, conception date)	88 cases (CL/P) 651 controls	One month pregestation + 1st trimester	59/88 (67) 1–6 times/wee k: 31/88 (35.2) >6 times: 28/88 (32)		348/651 (54) 1–6 times/week 234/651 (35.9) >6 times: 114/651 (17.5)	CL/P: 2 (1.2–3.4) Maternal occupation, fever and flu pregestation, child sex	a	a
**Li *et al* (2011)[23]**	Study: College Stomatology, West China Control: Women's and Children's Hospital, West China, **China**	Study: 2005–2008 Control: 2006–2007		162 cases 304 control		69/162 (42.6)		54/204 (17.4)		a	a
**Li *et al* (2008)[34]**	**Norway**	1996–2001	Matched case-control (time) random selected control	573 cases 763 controls	1st trimester	90/334 (26.9)	1st trimester CL/P: 58/210 (27.6) CP: 32/196 (163)	106/520 (20.4)	CLP: 1.59 (1.02–2.47) CP: 1.05 (0.55–2) Maternal education, occupation, alcohol intake, folic acid, supplement, diet and multivitamins, paternal income, child date of birth	239/432 (55.3)	243/763 (31.8)
**Little *et al* (2004)[8]**	Scotland, Manchester, Merseyside **UK**	1997–2000	Population-based Matched case-control (sex, date of birth, region)	190 cases 248 controls	1st trimester	67/110 (60.9)	1st trimester CL/P: 40.76 (52.6) CP 27/78 (34.6)	111/189 (58.7)	1 (0.6–1.6) Child sex, season of birth maternal education, ethnicity	80/190 (42.1)	59/248 (23.8)
**Mirilas *et al* (2011)[9]**	Pediatric Surgery Department, **Greece**	2004 & 2009	Residency Matched case-control	35 case control 35 matched (place)	One year pregestation or 1st trimester	34/35 (97.1)	CL/P: 16/35 (45.7)	25/35 (71.4)		15/35 (42.9)	20/35 (57.3
					One Year Pregestation + 1st trimester	16/35 (45.7)		11/35 (31.4)		9/53 (17)	7/35 (20)
					Pregestation	18/35 (51.4)		14/35 (40)			
**Taghafi *et al* (2012)[25]**	Bahrami Hospital, Tahran, **Iran**	2005–2010	Hospital base Case-control	300 cases 300 controls	Three month pregestation + 1st trimester	113/300 (37.7)	CL/P 113/300 (37.7)	80/300 (26.7)	0.613 (0.43–0.87) Child sex, maternal age, education, socioeconomic state, iron exposure, vitamin use, mediation smoking X-ray exposure, consanguinity	7/300 (2.3)	5/300 (2)
**Wang *et al* (2009)[26]**	Thirteen districts and countries, Shenyang, **China**	2000 to 2007	population-based control matched (gender, place, date of birth) (2 control for each case)		One month pregestation + 1st trimester	168/586 (28.7)		192/1172 (16.4)	2.05 (1.47–2.87) Maternal age and weight	12/586 (2)	16/1172 (1.4)
**Zhang *et al* (2010)[27]**	Centre for the Rehabilitation of Craniofacial Anomalies, Harbin Medical University. Harbin, **China**	2006–2009	Case-control Not matched	304 cases CLP 140 CP 77 CL 86 453 controls	One month pregestation + 1st trimester	224/323 (69.3)	CL: 79/106 (74.5) CLP: 96/140 (68.6) CP 49/77 (63.6)	169/454 (37.2)		14/300 (4.7)	6/545 (1.1)

a = missing information.

Shaw *et al* (1996) [10] reported positive associations between specific types of NSOFC and passive smoking, defined as when a non-smoking mother frequented, worked or lived in a place where others smoked within six feet of her (for CL/P OR: 2, 95% CI: 1.2 to 3.4; for CP OR:1.6, 95% CI: 0.7 to 3.4). However, as the study did not report the number of exposed cases and controls, it was excluded from meta-analysis, resulting in the meta-analysis being based on 14 studies. Wang *et al* (2009) [26] did not exclude smoking mothers from their analysis of maternal passive smoking. However, we included the study in our meta-analysis as this sample comprised very few mothers who reported smoking actively during pregnancy (2% in cases and 1.4% in control).

The definition of maternal passive smoking was similar in all 14 studies included in the meta-analysis. However, Li *et al* (2010) [23] defined passive smoking as the exposure of non-smoking mothers to at least one cigarette /week from a smoker in any place. Lie *et al*. (2008) [24] and Li *et al*. (2011) [22] defined it as non-smoking mother frequenting, working or living in a place where others smoked nearby, with Lie *et al*. (2008) [24] specifying a distance within two meters for at least two hours per day.

All studies measured maternal exposure to smoke during the three months of the first trimester apart from two studies which included only the first two months of the 1^st^ trimester [22, 25]. However, seven studies compared maternal exposure to smoke for the 1^st^ trimester combined with the pregestation period: one month pregestation [22, 26, 27]; three month pregestation [18, 20]; or one year pregestation [9, 21] (Table 1).

Honein *et al* (2007) [18] and Mirilas et al (2011) [9] measured the relationship between NSOFC and maternal passive smoking exposure in the pregestation period alone (see Table 1). Honein et al (2007) [18] have furthered analyzed the relationship with NSOFC type and found a significant relationship for CP (OR: 2.3, 95% CI: 1 to 5.3) but not with CL/P (OR: 1.3, 95% CI: 0.5 to 3.1).

The intensity of smoking was reported by two studies but with different measurement methods [23, 27]. Li et al (2010) [23] measured the number of maternal smoking exposure in hour per week, while Zhang et al (2011) [27] measured the number of hours of maternal exposure any time during one month pregestation and through the 1^st^ trimester.

### Meta-Analyses

Fig. 2 shows the forest plot for the relationship between maternal passive smoking and having an infant with NSOFC in the fourteen studies with complete information on the frequency of maternal exposure to passive smoking.

There was a significant relationship between passive maternal smoking and NSOFC. The risk of having an infant with NSOFC was doubled following maternal exposure to environmental tobacco (OR: 2.11, 95% CI: 1.54 to 2.89). When studies with ORs greater than 3 were excluded, the relationship continued to be significant; after excluding the study of Mirilas et al (2011) [9] the OR was 2.04 (95% CI: 1.49 to 2.8); after excluding that of Jia et al (2011) [19] in addition to that of Mirilas et al (2011) [9] the OR was 1.8 (95% CI: 1.4 to 2.32); after additionally excluding Zhang et al (2011) [27], the OR was 1.66 (95% CI: 1.34 to 2.06); and after exclusion of all studies with an OR greater than 3, the OR dropped further to 1.55 (95% CI: 1.28 to 1.87).

**Fig 3:**
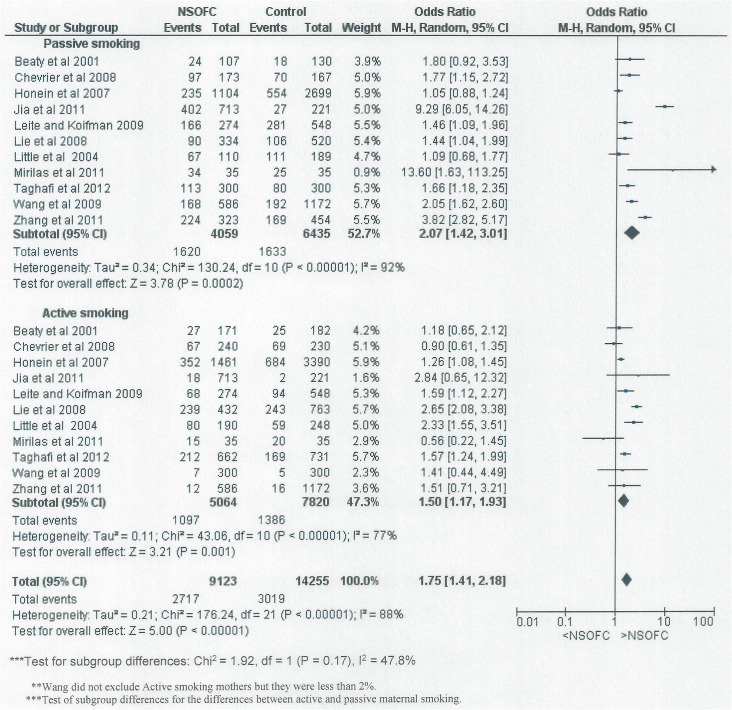
Forest plot for meta-analysis of the association between maternal passive smoking and the risk of having an infant with NSOFC, comparing the different types of maternal smoking exposure (active and passive).

Fig. 3 shows the forest plot for the relationship between maternal smoking and having an infant with NSOFC in studies that reported passive smoking and active smoking. Maternal active smoking and passive smoking was reported by eleven studies and found to be significantly related to NSOFC; OR: 2.07, 95% CI: 1.42 to 3.01 for passive smoking; and OR: 1.5, 95% CI: 1.17 to 1.93 for active smoking. Although the OR = 1.5 for active smoking was less than the OR = 2.07 for maternal passive smoking, the difference was not statistically significant (P = 0.17).

**Fig 4:**
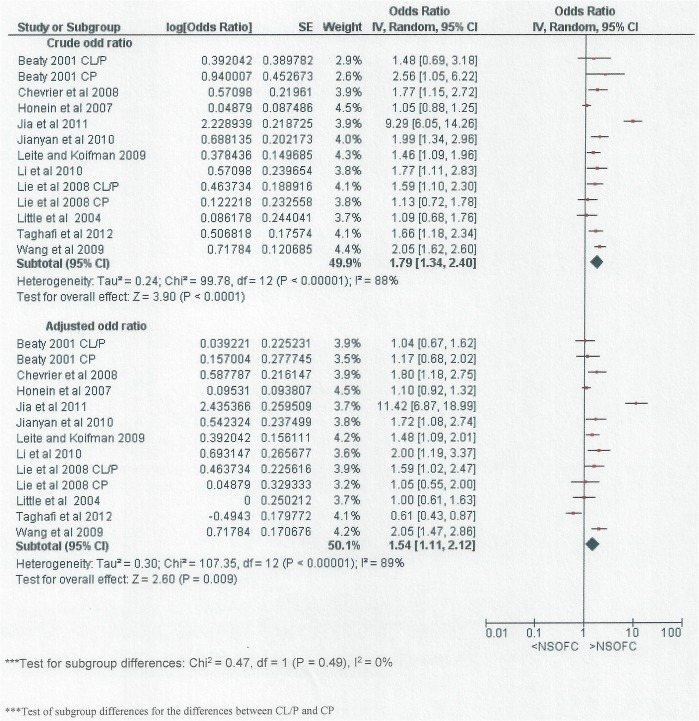
Forest plot for meta-analysis showing the crude and reported adjusted OR for the association between maternal passive smoking and NSOFC.

Fig. 4 included studies that reported adjusted OR for the association between NSOFC and passive smoking. Both meta-analysis; for the crude OR and the reported adjusted OR found a significant relationship between NSOFC and passive smoking (OR: 1.79, 95% CI: 1.34 to 2.4 and OR: 1.54, 95% CI: 1.11 to 2.12) respectively. In addition, there were no significant differences between the two meta-analyses (P = 0.49). The factors for which adjustment was made in each study are listed in Table 1 and differed between studies.

**Fig 5:**
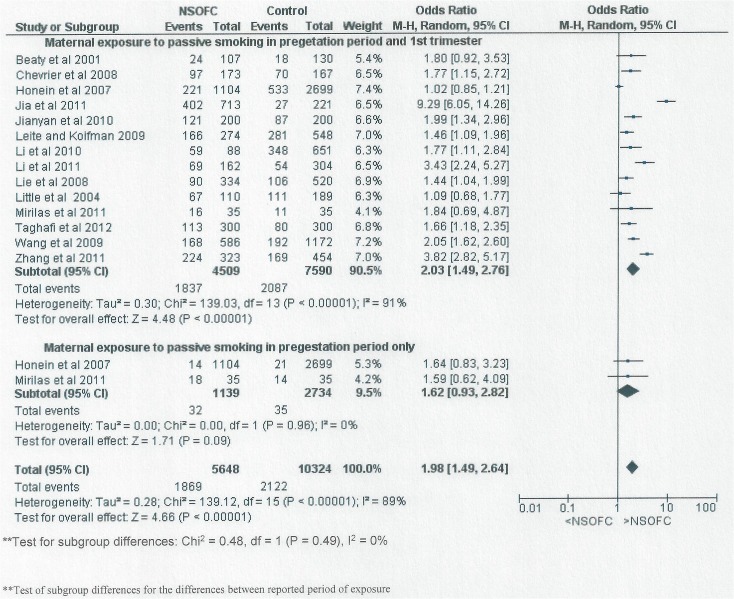
Forest plot for meta-analysis of the association between NSOFC and maternal passive smoking exposure in the 1st trimester including or not including the pregestation period compared to maternal exposure prior to pregnancy period alone.

Fig. 5 shows forest plot for meta-analysis of the association between maternal passive smoking exposure period and NSOFC. The period of maternal exposure was divided into two groups; studies reporting maternal passive smoking exposure during the 1^st^ trimester including or not including the pregestation period and studies reporting maternal passive smoking exposure prior to pregnancy alone. NSOFC was significantly associated with maternal exposure to passive smoking during the first trimester period including or not including pregestation period (OR: 2.03, 95% CI: 1.49 to 2.76). However, no significant relationship was found for maternal exposure prior to pregnancy alone (OR 1.62, 95% CI: 0.93 to 2.82). The difference between the two periods was not statistically significant (P = 0.49).

**Fig 6:**
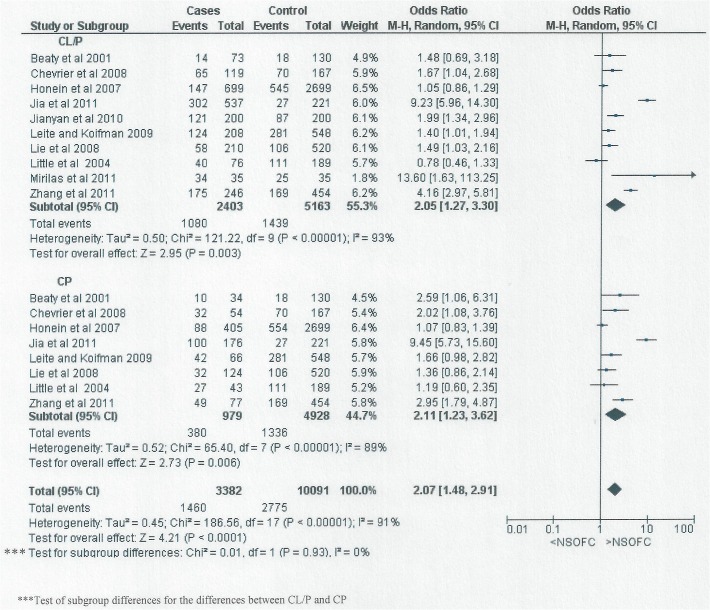
Forest plot for meta-analysis of the association between NSOFC phenotype (CL/P and CP) and maternal passive smoking.

Fig. 6 shows the relationship between passive smoking and the different types of NSOFC; CL/P and CP. The risk of having an infant with either CL/P or CP associated with passive smoking was approximately doubled (for CL/P OR: 2.05, 95% CI: 1.27 to 3.3; for CP OR: 2.11, 95% CI: 1.23 to 3.62).

**Fig 7:**
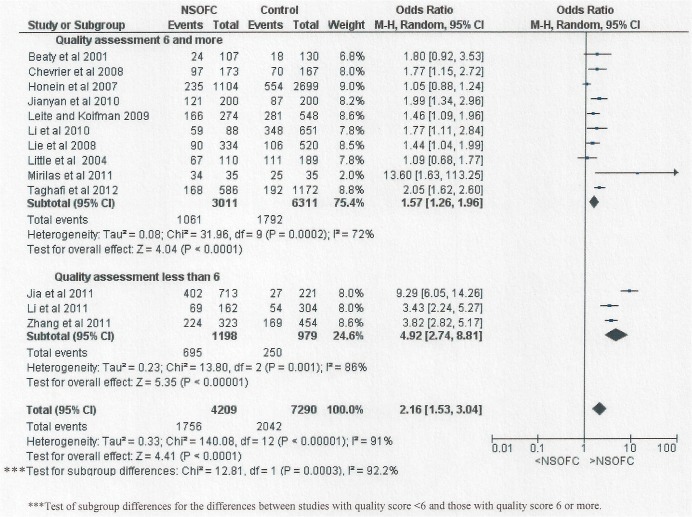
Forest plot for meta-analysis of the association between maternal passive smoking and NSOFC according to region.

Papers assessing the relationship between maternal smoking and having an infant with NSOFC were also sub-grouped according to region (China, United State and Europe, and other countries) (Fig. 7). There was a significant difference between the three regions (P = 0.01) with a higher OR (OR: 3.08, 95% CI: 1.96 to 4.87) for China than the other two regions. However, high heterogeneity remained between studies within China, and within the US and European group.

**Fig 8:**
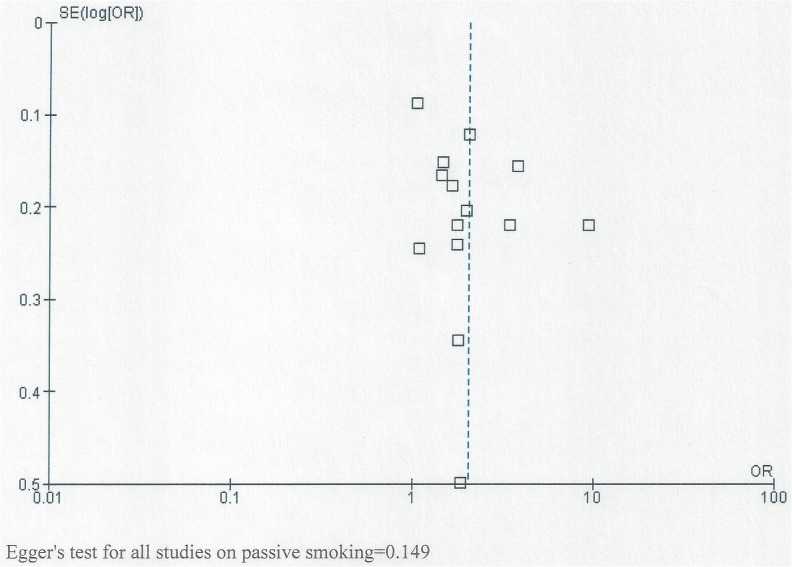
Forest plot for meta-analysis of the association between maternal passive smoking and NSOFC according to study quality (NOS scale).

The magnitude of association between maternal passive smoking and NSOFC was significantly higher (P = 0.0003) in the studies assessed as of “fair” or “poor” quality than in the other studies. However, differences in study quality and risk of bias do not account for the substantial heterogeneity of effect between studies (Fig. 8). Each meta- analyses showed significant heterogeneity with I^2^ more than 75% for the majority of subgrouping.

### Assessing risk of bias

Fig. 8 shows the included studies distributed according to NOS risk of bias scores. Out of the 14 studies, 11 had a low risk of bias score [8, 9, 16–18, 20, 21, 23–26] (OR: 1.57, 95% CI: 1.26 to 1.96); while three of the studies were rated being at moderate to high risk [19, 22, 27] (OR: 4.92, 95% CI: 2.74 to 8.81). The main reason for the lower NOS in some studies was lack of comparability and matching. There was a significant difference in the maternal passive smoking risk OR between these two groupings (P = 0.0003).

### Sensitivity analysis

The sensitivity analysis demonstrated stability and reliability of the meta-analyses results through consistency of meta-analyses results, between different study subgroupings; the significant relationships between maternal passive smoking and NSOFC persisted in all of the situations evaluated (Figs. 3, 4, 5, 6 and 7).

### Meta regression

Table 2 shows that the association between passive smoking and NSOFC varies by study quality, region but not cleft type. The magnitude of the association is lower in studies appraised as of good quality than in other studies and higher for Asian studies.

**Table 2:** Univariate meta-regression analysis relating cleft type and study quality to effect size estimates of the relation between passive smoking and NSOFC.

Variable	Coefficient	Standard error	p value	Relative odds ratio (95% CI)	Tau-squared
**Constant**	0.579	0.636	0.379	----	
**Cleft type**	0.132	0.411	0.752	1.14 (0.47 to 2.77)	0.566
**Constant**	1.579	0.188	0.000	----	
**Quality**	−1.144	0.214	0.000	0.32(0.20 to 0.51)	0.067
**Constant**	0.221	0.067	0.007	----	
**Region**	0.407	0.134	0.011	1.50(1.12 to 2.02	0.090

### Evaluation of small study effects

**Fig 9:**
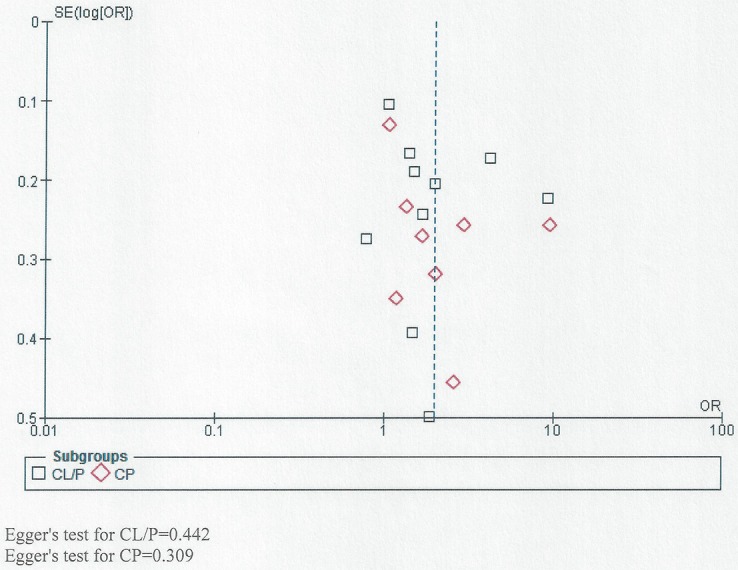
Funnel plot for studies showing the relationship between passive smoking and both CL/P and CP.

Fig. 9 shows the funnel plots for all studies together assessing the relationship between NSOFC and passive smoking. It also shows studies assessing the relationship between passive smoking and NSOFC phenotype (CL/P and CP). Though the graph does not have the shape of a funnel, it is almost symmetrical around the central line, indicating absence of small study effect. No statistically significant small study effect was detected by Egger’s test (to further assess small study effect), either for all studies together and for studies of specific cleft types (P>0.05).

## Discussion

This systematic review and meta-analysis found around a twofold increase in the risk of NSOFC associated with environmental tobacco exposure. Not all studies adjusted for potential confounding factors, and in those that did, the covariates differed between studies. In the studies that presented both crude and adjusted estimates of effect, there was a modest attenuation of the magnitude of association (OR reduced from (1.79 to 1.54) and it was noteworthy that the magnitude of association in studies appraised as having a low risk of bias was about 1.5. Overall, the magnitude of association was similar between CL/P and CP, but there was substantial heterogeneity between studies.

Prevention and protecting against having an infant with NSOFC could be possible through understanding the associated risk factors. In 2014, the Surgeon General considered the evidence in the literatures to be sufficient to infer a causal relationship between maternal smoking in early pregnancy and orofacial clefts [6].

However, the effects of passive smoking have not been fully evaluated and a single study would provide insufficient evidence [28]. In addition, passive smoke could have a more potent adverse effect on infants in a domestic environment as pregnant women and nursing mothers might be unaware of its existence or its importance as a risk factor. In some countries, smoking has been prohibited in enclosed public places to protect non-smoking individuals from passive smoking. However, many other countries, both developing and some developed countries, have not introduced smoking restriction legislation [29]; and mothers may be exposed to passive smoke in the domestic environment due to heating and cooking as well as tobacco smoke.

Environmental smoking exposure is difficult to measure. The main methods of assessment are self-report and biochemical assays of nicotine or cotinine [30]. All studies included in this meta-analysis used self-reported questionnaires. Kvalvik *et al* (2012) [30] validated maternal self-reported tobacco use during pregnancy with plasma cotinine in the Norwegian Mother and Child Cohort Study. They concluded that self-reported tobacco use was a valid marker for tobacco smoke exposure including passive smoking. In addition, Salmasi *et al* (2010) [31] compared the results of the association between perinatal outcomes and maternal self-reported exposure to passive smoking and biochemical analysis (2–10 ng/ml of cotinine) and found similar findings with both methods of exposure assessment.

Eleven out of the 14 studies included in the meta-analysis presented data on maternal active smoking as well as on the effects of passive smoking in non-smoking mothers. In aggregate, passive smoking increased the risk of NSOFC (OR: 2.07, 95% CI: 1.42 to 3.01) more than active smoking (OR: 1.5, 95% CI: 1.17 to 1.93), although this difference was not statistically significant (P = 0.17) and there was considerable heterogeneity between studies. Given that active smokers are exposed both to direct inhalation and to sidestream smoke, a stronger association for passive smoking than for active smoking is unexpected. A possible explanation for this finding could be under-reporting of active smoking as the studies rely on self-report and active smoking mothers may be compelled to under-report their smoking because of the associated stigma. This sense of shame would not influence reporting of the passive smoking reports to the same extent. Another explanation or contributory factor could be that duration/ dose response might influence the high association for passive smoking compared to active smoking; mothers may be exposed to passive smoke under occupational circumstances which would lead to a longer duration of exposure. However, there was insufficient detail in the reports to be able to extract this level of data.

Such a difference might be due to active smoking mothers tending to stop smoking when they are pregnant but being unaware that environmental tobacco exposure is harmful to the health of the developing infant. Therefore, public health awareness and further studies on passive smoking are important.

Our search focused on manuscripts of passive smoking studies. Whilst active smoking results are reported in some of these passive smoking studies’ papers, our results do not represent a complete picture of the active smoking literature. However, active smoking has only been used for the purpose of comparison in assessing the passive smoking effect In order to probe the association between passive smoking and NSOFC further, in the eleven studies that reported adjusted OR, we compared the adjusted OR and crude OR. Although the differences between the estimates were not formally significant (P = 0.49), there was some attenuation of the OR by adjustment.

We found a higher risk of association between NSOFC and maternal passive smoking in the 1^st^ trimester including or not including the pregestation period (OR: 2.03, 95% CI: 1.49 to 2.76) compared to those who were exposed to environmental tobacco only in the period prior to pregnancy (OR: 1.62, 95% CI: 0.93 to 2.82) but the difference between the two periods is not statistically significant (P = 0.49). This could indicate that if mothers were exposed to passive smoking prior to pregnancy, there is still an opportunity to protect their embryo from NSOFC through avoiding passive smoking. However, this finding needs more investigation as there were only two studies that reported maternal passive smoking exposure prior to pregnancy.

The magnitude of association (OR = 4.92) in the three studies with a NOS lower than 6 was significantly higher odds ratio than for the studies with a NOS scoring 6 or more. These studies were all carried out in China, which could in part explain the significant difference between the three different regions (Fig. 7), with the higher OR in studies carried out in China (OR: 3.08) compared to other regions (OR: 1.39 for the studies in Europe and US; and OR: 1.54 for the studies in Iran and Brazil). It is noteworthy that in all but one [23] of the Chinese studies, the prevalence of reported passive smoking in control mothers was substantially lower than has been reported in pregnant women [32] or women of reproductive age [33, 34] in large surveys in China.

The two categories associated with low NOS study scoring were "exposure ascertainment" and "comparability". The ascertainment of exposure was affected because information on smoking was gathered through questionnaire or interview, which is not usually considered to provide optimal information on smoking, although there is evidence that suggests that self-reported passive smoking gives information of comparable quality to cotinine assessment [30]. The comparability between cases and controls in terms of minimizing the effects of potential confounding by means of matching and/or adjustment for confounding variables was also limited in studies with low quality (Fig. 3). Therefore, we strongly advise careful consideration of the comparability of source populations in recruiting cases and controls in future studies.

Further research on the intensity, duration of exposure and agreement on standardized methods for recording and reporting will aid further investigation of this environmental hazard.

Tobacco use is rapidly increasing among women of reproductive age in many countries because they are actively targeted by tobacco marketing campaigns [35, 36] and this would be likely to result in an increase in the prevalence of exposure to environmental tobacco smoke. In the U.S., a decline in the prevalence of exposure to passive smoking from the late 1980s has leveled off since about 2002 [37]. There also continues to be substantial exposure to passive smoking in Canada [38]. In England, the impact of smoke-free legislation on exposure to passive smoking was greater than the underlying long-term decline in exposure, demonstrating a positive effect of legislation [39]. An increased risk of cleft palate associated with passive smoking has been mentioned in a paper making a case for a worldwide ban on smoking in public places [40]. Therefore, we suggest that the results of the present meta-analysis provide a more solid basis to argue for interventions to minimize exposure to environmental tobacco smoke in policy making fora and in health promotion initiatives.

## Conclusion

In studies that adjust for potential confounding and/or are adjudged to have low risk of bias, maternal passive smoking exposure is associated with approximately a 1.5 fold increase in the risk of having an infant with NSOFC. There is marked heterogeneity between studies, which is not explained by differences in the distribution of cleft type, adjustment for covariates, difference in regions, or study quality. This thorough meta-analysis provides further evidence to argue for interventions to minimize exposure to environmental tobacco smoke in policy making fora and in health promotion initiatives.

## Supporting Information

S1 PRISMA Checklist (DOC)

S1 Fig Search strategy for systematic review assessing the relationship between passive smoking and non-syndromic oral cleft. (PDF)

S2 Fig Quality table and coding manual. (PDF)

S1 Table Data extraction form. (XLSX)
