# Supplementary material for: Passive Smoking in the Etiology of Non-Syndromic Orofacial Clefts: A Systematic Review and Meta-Analysis
Source: PLoS One. 2015 Mar 11;10(3):e0116963. doi: 10.1371/journal.pone.0116963 (PMC4356514; doi:10.1371/journal.pone.0116963)
Supplement: S1 Fig — (PDF) [file pone.0116963.s002.pdf]

S1_Search strategy for systematic review assessing the relation between passive smoking and non-syndromic orofacial cleft

Search engine:

Scopus, PubMed, Google literature.

Key words listed in table 1

Inclusion criteria:

- Case-control studies comparing passive smoking with NSOFC
- Cross sectional or cohort studies relating passive smoking with NSOFC
- Time limitation 1995-2013

Exclusion criteria:

- Cross sectional studies or cohort studies that do not include a control group
- Studies discussing other type of smoking and etiological factors.
- Studies that discussed congenital abnormalities as a whole and did not specify non-syndromic OFC cases in the analysis.
- No language exclusion

Table listing the keywords used in the search with the number of article found in each search engine:

Key Words	No of Articles
Scopus	
TITLE-ABS-KEY(cleft lip)	17,433
TITLE-ABS-KEY-AUTH(cleft palate)	25,229
TITLE-ABS-KEY-AUTH(orofacial cleft)	1,002
TITLE-ABS-KEY-AUTH(tobacco smoke pollution)	11,222
TITLE-ABS-KEY-AUTH(passive smoking)	9,335
TITLE-ABS-KEY-AUTH(second hand smoking)	1,401
TITLE-ABS-KEY-AUTH(environmental tobacco smoke pollution)	5,394
TITLE-ABS-KEY(cleft PRE/10 lip)	17,008
TITLE-ABS-KEY-AUTH(smoking)	270,416

(TITLE-ABS-KEY(cleft lip)) OR (TITLE-ABS-KEY-AUTH(cleft palate)) OR (TITLE-ABS-KEY-AUTH(orofacial cleft))	28,473
TITLE-ABS-KEY-AUTH(passive smoking)) OR (TITLE-ABS-KEY-AUTH(tobacco smoke pollution)) OR (TITLE-ABS-KEY-AUTH(environmental tobacco smoke pollution))	15,468
(TITLE-ABS-KEY-AUTH(smoking)) OR (TITLE-ABS-KEY-AUTH(passive smoking)) OR (TITLE-ABS-KEY-AUTH(secondhand smoking)) OR (TITLE-ABS-KEY-AUTH(tobacco smoke pollution)) OR (TITLE-ABS-KEY-AUTH(environmental tobacco smoke pollution))	272,348
((TITLE-ABS-KEY-AUTH(smoking)) OR (TITLE-ABS-KEY-AUTH(passive smoking)) OR (TITLE-ABS-KEY-AUTH(secondhand smoking)) OR (TITLE-ABS-KEY-AUTH(tobacco smoke pollution)) OR (TITLE-ABS-KEY-AUTH(environmental tobacco smoke pollution))) AND ((TITLE-ABS-KEY(cleft lip)) OR (TITLE-ABS-KEY-AUTH(cleft palate)) OR (TITLE-ABS-KEY-AUTH(orofacial cleft)))	329
TITLE-ABS-KEY(cleft lip)) OR (TITLE-ABS-KEY-AUTH(cleft palate)) OR (TITLE-ABS-KEY-AUTH(orofacial cleft))) AND ((TITLE-ABS-KEY-AUTH(passive smoking)) OR (TITLE-ABS-KEY-AUTH(tobacco smoke pollution)) OR (TITLE-ABS-KEY-AUTH(environmental tobacco smoke pollution)))	37
**Total**	**366**
PubMed	
(("cleft lip"[MeSH Terms] OR ("cleft"[All Fields] AND "lip"[All Fields]) OR "cleft lip"[All Fields]) OR ("cleft palate"[MeSH Terms] OR ("cleft"[All Fields] AND "palate"[All Fields]) OR "cleft palate"[All Fields]) OR (orofacial[All Fields] AND cleft[All Fields])) AND (("tobacco smoke pollution"[MeSH Terms] OR ("tobacco"[All Fields] AND "smoke"[All Fields] AND "pollution"[All Fields]) OR "tobacco smoke pollution"[All Fields]) OR ("tobacco smoke pollution"[MeSH Terms] OR ("tobacco"[All Fields] AND "smoke"[All Fields] AND "pollution"[All Fields]) OR "tobacco smoke pollution"[All Fields] OR ("environmental"[All Fields] AND "tobacco"[All Fields] AND "smoke"[All Fields] AND "pollution"[All Fields]) OR "environmental tobacco smoke pollution"[All Fields]) OR ("tobacco smoke pollution"[MeSH Terms] OR ("tobacco"[All Fields] AND "smoke"[All Fields] AND "pollution"[All Fields]) OR "tobacco smoke pollution"[All Fields] OR ("passive"[All Fields] AND "smoking"[All Fields]) OR "passive smoking"[All Fields]) OR ("smoking"[MeSH Terms] OR "smoking"[All Fields]))	189
(("cleft lip"[MeSH Terms] OR ("cleft"[All Fields] AND "lip"[All Fields]) OR "cleft lip"[All Fields]) OR ("cleft palate"[MeSH Terms] OR ("cleft"[All Fields] AND "palate"[All Fields]) OR "cleft palate"[All Fields]) OR (orofacial[All Fields] AND cleft[All Fields])) AND (("tobacco smoke pollution"[MeSH Terms] OR ("tobacco"[All Fields] AND "smoke"[All Fields] AND "pollution"[All Fields]) OR "tobacco smoke pollution"[All Fields]) OR ("tobacco smoke pollution"[MeSH Terms] OR ("tobacco"[All Fields] AND "smoke"[All Fields] AND "pollution"[All Fields]) OR "tobacco smoke pollution"[All Fields] OR ("environmental"[All Fields] AND	26

"tobacco"[All Fields] AND "smoke"[All Fields] AND "pollution"[All Fields]) OR "environmental tobacco smoke pollution"[All Fields]) OR ("tobacco smoke pollution"[MeSH Terms] OR ("tobacco"[All Fields] AND "smoke"[All Fields] AND "pollution"[All Fields]) OR "tobacco smoke pollution"[All Fields] OR ("passive"[All Fields] AND "smoking"[All Fields]) OR "passive smoking"[All Fields]))	
**Total**	**215**
Google Scholar	
(cleft lip OR cleft palate OR orofacial cleft) AND (passive smoking OR tobacco smoke pollution OR environmental tobacco smoke pollution)	425
**Total**	**425**
**Total articles**	**1006**
