# Supplementary material for: Passive Smoking in the Etiology of Non-Syndromic Orofacial Clefts: A Systematic Review and Meta-Analysis
Source: PLoS One. 2015 Mar 11;10(3):e0116963. doi: 10.1371/journal.pone.0116963 (PMC4356514; doi:10.1371/journal.pone.0116963)
Supplement: S2 Fig — (PDF) [file pone.0116963.s003.pdf]

S2: Quality scoring for included papers using Newcastle-Ottawa Scale (NOS)

S2: Quality scoring for studies included in the meta-analysis. For each paper a score was given consisting of; a letter (a, b or c) that stands for which of the NOS quality coding item list describe the paper more; and a number (0 or 1) which is the score value for this description.

	Reference	Case definition	Representation	Control selection	Control definition	Comparability	Exposure ascertainment	Same method of ascertainment	Response rate	Total score
1	Beaty et al (2001)	a1	a1	a1	a1	a1b1	a1	a1	a1	9
2	Chevrier et al (2008)	a1	a1	b0	a1	a1 b1	c0	a1	a1	7
3	Honein et al (2007)	a1	a1	a1	a1	a1	c0	a1	a1	7
4	Jia et al (2011)	a1	a1	b0	a1	a1	c0	a1	c0	5
5	Jianyan et al (2010)	a1	a1	b0	a1	a1	c0	a1	a1	6
6	Leite and Koifman (2009)	b0	a1	a1	a1	a1b1	c0	a1	c0	6
7	Li et al (2010)	a1	a1	b0	a1	a1	c0	a1	a1	6
8	Li et al (2011)	a1	a1	b0	a1	a1	c0	a1	c0	5
9	Lie et al (2008)	a1	a1	a1	b0	a1	a1	a1	a1	7
10	Little et al (2004)	a1	a1	a1	a1	a1b1	a1	b0	a1	8
11	Mirilas et al (2011)	a1	a1	b0	a1	a1	a1	a1	a1	7
12	Taghavi et al (2012)	a1	a1	a1	a1	a1	c0	a1	a1	7
13	Zhang et al (2011)	c0	a1	b0	a1	0	0	a1	b0	3

NOS CODING MANUAL FOR CASE-CONTROL STUDIES

SELECTION

1) Is the Case Definition Adequate?

a) Requires some independent validation (e.g. >1 person/record/time/process to extract information, or reference to primary record source such as x-rays ormedical/hospital records)
b) Record linkage (e.g. ICD codes in database) or self-report with no reference to primary record
c) No description

2) Representativeness of the Cases

a) All eligible cases with outcome of interest over a defined period of time, all cases in a defined catchment area, all cases in a defined hospital or clinic, group of hospitals, health maintenance organization, or an appropriate sample of those cases (e.g. random sample)
b) Not satisfying requirements in part (a), or not stated.

3) Selection of Controls

This item assesses whether the control series used in the study is derived from the same population as the cases and essentially would have been cases had the outcome been present.
a) Community controls (i.e. same community as cases and would be cases if had outcome)
b) Hospital controls, within same community as cases (i.e. not another city) but derived from a hospitalized population
c) No description

4) Definition of Controls

a) If cases are first occurrence of outcome, then it must explicitly state that controls have no history of this outcome. If cases have new (not necessarily first) occurrence of outcome, then controls with previous occurrences of outcome of interest should not be excluded.
b) No mention of history of outcome

COMPARABILITY

1) Comparability of Cases and Controls on the Basis of the Design or Analysis

A maximum of 2 stars can be allotted in this category

Either cases and controls must be matched in the design and/or confounders must be adjusted for in the analysis. Statements of no differences between groups or that differences were not statistically significant are not sufficient for establishing comparability.
Note: If the odds ratio for the exposure of interest is adjusted for the confounders listed, then the groups will be considered to be comparable on each variable used in the adjustment.
There may be multiple ratings for this item for different categories of exposure (e.g. ever vs. never, current vs. previous or never)
Age = *****, Other controlled factors = *****

EXPOSURE

1) Ascertainment of Exposure

Allocation of stars as per rating sheet

2) Non-Response Rate

Allocation of stars as per rating sheet
